# Mild behavioral impairment domains are longitudinally associated with pTAU and metabolic biomarkers in dementia‐free older adults

**DOI:** 10.1002/alz.13902

**Published:** 2024-06-14

**Authors:** Emmanuel Gonzalez‐Bautista, Marie Momméja, Adelaïde de Mauléon, Zahinoor Ismail, Bruno Vellas, Julien Delrieu, Maria E. Soto Martin

**Affiliations:** ^1^ Research and Clinical Alzheimer's Disease Center CMRR CHU Toulouse IHU HealthAge Toulouse France; ^2^ Maintain Aging Research team CERPOP Université de Toulouse Inserm, Université Paul Sabatier Toulouse France; ^3^ Departments of Psychiatry Clinical Neurosciences Community Health Sciences, and Pathology Hotchkiss Brain Institute and O'Brien Institute for Public Health University of Calgary Calgary Alberta Canada; ^4^ Clinical and Biomedical Sciences Faculty of Health and Life Sciences University of Exeter Exeter UK

**Keywords:** homocysteine, mild behavioral impairment, neuropsychiatric symptoms, tau protein

## Abstract

**BACKGROUND:**

The mechanisms linking mild behavioral impairment (MBI) and Alzheimer's disease (AD) have been insufficiently explored, with conflicting results regarding tau protein and few data on other metabolic markers. We aimed to evaluate the longitudinal association of the MBI domains and a spectrum of plasma biomarkers.

**METHODS:**

Our study is a secondary analysis of data from NOLAN. The longitudinal association of the MBI domains with plasma biomarkers, including pTau181, was tested using adjusted linear mixed‐effects models.

**RESULTS:**

The sample comprised 359 participants (60% female, mean age: 78.3, standard deviation: 0.3 years). After 1 year, the MBI domain of abnormal perception was associated with steeper increases in plasma pTau181. Abnormal perception, decreased motivation, and impulse dyscontrol were associated with homocysteine or insulin dysregulation.

**DISCUSSION:**

Apart from the association with plasma pTau181, our results suggest that MBI might also represent metabolic dysregulation, probably contributing to dementia transition among older adults with subjective cognitive decline or mild cognitive impairment.

**Highlights:**

- Mild behavioral impairment (MBI) psychosis was associated with steeper increases in plasma p.
- pTau could be a pharmacological target to treat agitation and psychosis symptoms.
- MBI domains were linked to metabolic dysregulation involving insulin and homocysteine.

## 1 BACKGROUND

Agitation and other neuropsychiatric symptoms (NPS) are often the most disturbing aspects of Alzheimer's disease (AD) dementia for the patients and their caregivers.^1^ However, management of NPS is not straightforward as few drugs are approved to treat NPS in dementia. Late‐onset NPS may have a different pathogenesis than psychiatric diseases of remote onset, which may develop independently of cognitive impairment or dementia.^2^ Bridging the pathophysiological knowledge gap on the link between NPS and dementia may lead to finding new therapeutic targets that can relieve NPS and possibly attenuate dementia risk and delay its onset.

The study of NPS in dementia has been fostered by the recognition of mild behavioral impairment (MBI), marked by later‐life emergent and persistent NPS in dementia‐free older persons, ranging from cognitively unimpaired to subjective cognitive decline (SCD) to mild cognitive impairment (MCI).^3^, ^4^ MBI represents a high‐risk group for incident cognitive decline and dementia, reflecting preclinical or prodromal disease in some. MBI can be a prodromal marker of dementia and a predictor of faster progression to dementia in SCD and MCI populations. Specifically, MBI is intended to identify behavioral risk, which is best interpreted in conjunction with cognitive risk, as MBI rates of progression to dementia differ by concurrent cognitive status (cognitively normal, SCD, or MCI).^5^, ^6^, ^7^

The neurodegenerative mechanisms behind MBI are not fully understood; however, studies have explored the association of MBI with Alzheimer's disease (AD)‐related biomarkers. Regarding amyloid beta (Aβ), significant associations have been found between MBI and amyloid positron emission tomography (PET) among cognitively normal Translational Biomarkers in Aging and Dementia (TRIAD) participants,^8^ between MBI and cerebrospinal fluid (CSF) amyloid among MCI participants in Alzheimer's Disease Neuroimaging Initiative (ADNI) and Memento,^9^ and between MBI and plasma Aβ41/42 ratio in mixed dementia‐free ADNI participants.^10^ Regarding tau, no significant association was found between MBI and PET tau binding in cognitively normal TRIAD participants^8^ or between MBI and PET tau binding in mixed dementia‐free ADNI participants.^11^ Significant associations were found between MBI and plasma p‐tau in mixed dementia‐free ADNI participants^12^ and between MBI and CSF p‐tau in MCI participants in ADNI and Memento.^13^

**RESEARCH IN CONTEXT**

1. **Systematic review**: We conducted a literature review utilizing PubMed looking at studies linking neuropsychiatric symptoms (NPS) to a spectrum of neurodegeneration and metabolic biomarkers to bridge the knowledge gap on mechanisms linking MBI and AD.
2. **Interpretation**: Our findings suggest that, besides tauopathy, metabolic disturbances relating homocysteine and insulin could be mechanisms leading to MBI or accelerating the progression of subjective cognitive decline or mild cognitive impairment to dementia.
3. **Future directions**: The manuscript sheds light on the association between MBI and plasma pTau181 by identifying that the MBI domains of abnormal perception and social inappropriateness are linked to steeper increases of plasma p‐tau. If further studies strengthen the evidence on the MBI–pTau181 association, pTau‐binding antibodies could be a pharmacological option to treat agitation and psychosis symptoms in the future. In addition, our results open the possibility of continuing to explore the metabolic processes that may be involved in preclinical dementia, notably clarifying the role of homocysteine and insulin.

Studies have also assessed MBI links with tau in relation to Aβ. MBI was found to be associated with both CSF p‐tau and PET tau binding in cognitively normal amyloid+ Translational Biomarkers in Aging and Dementia‐2 (BIOFINDER‐2) participants.^14^ Most recently, Aβ status was found to moderate the association between MBI status and PET tau uptake in brain regions affected early in AD, in mixed dementia‐free ADNI participants.^15^ The inconsistency in tau results is likely due to different operationalizations of MBI, especially regarding duration of symptoms. For neurodegeneration, a significant association was found between MBI and plasma neurofilament light chain^16^ in mixed dementia‐free ADNI participants, and between MBI and CSF total tau in ADNI and Memento participants with MCI.^9^ Psychosis has been linked to tau in AD,^17^ but for MBI domains, results have yet to emerge. The association of plasma p‐tau with individual MBI domains in SCD and MCI populations is also yet to be assessed.

Metabolic and inflammatory biomarkers have also been linked to NPS in populations with heterogeneous cognitive status, such as the glial fibrillary acidic protein (GFAP),^18^, ^19^ insulin,^20^, ^21^ omega‐3,^22^, ^23^ vitamin D,^17^, ^24^ homocysteine,^25^, ^26^ and ferritin/transferrin.^27^, ^28^ However, studies correlating these metabolic and inflammatory biomarkers with NPS in SCD and MCI populations are scarce.

This study aimed to explore the longitudinal association of neurodegeneration, metabolic, and inflammatory biomarkers with the MBI (categorized by domains) from participants in the NOLAN study. Secondarily, we aimed to explore the interaction of the MBI domains and the biomarkers across cognitive function levels.

## 2 METHODS

### 2.1 Study design and population

Our study is a secondary analysis of data from NOLAN, a randomized control trial (RCT) aimed at evaluating the effect of a nutritional supplement on biomarkers and cognitive function among older adults. Participants were randomized 1:1 to receive either the intervention (oral nutritional blend) or placebo. The nutrient composition of the mix is provided as supplementary material 1.

NOLAN's methodological details have been published elsewhere.^29^ Briefly, the NOLAN study was conducted in community‐dwelling older adults recruited between 2016 and 2018, with follow‐up ending in 2019. Inclusion criteria were age ≥70 years, self‐reporting subjective memory complaints, and having a study partner to participate as a source of information. Exclusion criteria were taking vitamin B supplements in the past 3 months, taking ω‐3 polyunsaturated fatty acid supplements containing >200 mg of docosahexaenoic acid (DHA)/day over 6 months before inclusion, having difficulty with more than two basic activities of daily living (ADL) (Katz score <4),^30^ Mini–Mental State Examination (MMSE) score <24,^31^ or diagnosis of dementia. Detailed inclusion and exclusion criteria have been described elsewhere.^29^ Additionally, for our study purposes, we excluded three NOLAN participants who reported major psychiatric disorders (bipolar disorder *n* = 2, major depression *n* = 1). Participants underwent six in‐person visits, two before baseline and at 1, 6, and 12 months, with a mean drop‐out rate of 15.7%.

The NOLAN study (clinicaltrials.gov NCT03080675) was approved by the Advisory Committee for Protection of Persons South West and Overseas II (CPP SOOM II) and by the French Agency for the Safety of Medicines and Health Products (ANSM). All participants provided signed, informed consent.

Data pertinent to this current study were collected at baseline and 12 months.

### 2.2 Neuropsychiatric symptoms and mild behavioral impairment

A family member or close informant completed the Neuropsychiatric Inventory Questionnaire (NPI‐Q)^32^ at baseline and 12 months. The NPI‐Q evaluates the status and severity of 10 NPS (delusions, hallucinations, agitation/aggression, dysphoria/depression, anxiety, irritability, disinhibition, euphoria, apathy, aberrant motor behavior) and two neurovegetative domains (sleep and night‐time behavior and appetite/eating), each item with a severity scoring from 1 (mild) to 3 (severe). Following the ISTAART‐Alzheimer's Association (AA) research diagnostic criteria,^3^ we used a previously published approach to approximate the MBI‐C domains using the NPI‐Q items.^6^, ^33^ We used 10 of the NPS assessed by NPI‐Q to operationalize the five MBI domains as follows: decreased motivation (NPI‐Q apathy/indifference); emotional/affective dysregulation (NPI‐Q depression/dysphoria, anxiety, elation/euphoria); impulse dyscontrol (NPI‐Q agitation/aggression, irritability liability, aberrant motor behavior); social inappropriateness (NPI‐Q disinhibition); and abnormal perception or through content (NPI‐Q delusions, hallucinations).

*MBI domain status* was defined as present if at least one behavior comprising a specific domain was positive, absent otherwise. The MBI domains were introduced in the models as time‐varying to maximize the period sensitive to MBI detection.

*MBI domain severity* was defined as the arithmetical addition of the severity from the NPI‐Q items (eg, the severity of the MBI domain of abnormal perception may range from 0 to 6, given that it includes the two NPI‐Q items of delusions and hallucinations).

The NPI‐Q neurovegetative domains of sleep and appetite are not easily mapped onto the ISTAART‐AA MBI criteria. Of note, as the NPI‐Q has a reference range of 1 month, a shorter reference period was used to approximate MBI, which requires 66 months’ duration of new‐onset symptoms.

### 2.3 Biomarkers

Plasma samples were collected at baseline, 6 months, and 12 months of follow‐up, but pTau181 and GFAP were assessed only at baseline and 12 months. Plasma pTau181 using an in‐house Simoa method based on the AT270 (specific for the threonine‐181 phosphorylation site) and Tau12 (N‐terminal epitope 6–18 on human tau protein).^34^ Plasma GFAP concentration was measured using the commercially available GFAP Discovery assay (Quanterix, Billerica, MA, USA). All measurements were performed on an HD‐X Analyzer (Quanterix) with baseline and follow‐up samples from the same participant side by side on the assay plates by board‐certified laboratory technicians blinded to clinical information. Intra‐assay coefficients of variation were below 10%.

We also included other biomarkers measured at baseline and 12 months: the ω−3 index (ie, the sum of percentage docosahexaenoic acid and percentage eicosapentaenoic acid, expressed as the percentage of total erythrocyte membrane fatty acids),^35^ homocysteine (umol/L), insulin (pmol/L), 25 hydroxyvitamin D (ng/mL), ferritin (ng/mL), and transferrin (g/L).

### 2.4 Covariates

Age was estimated according to birthdate. Sex was self‐reported. Time was measured in months (baseline = 0, follow‐up = 12), APOE ε4 status (0 = negative, 1 = positive), education (years of formal instruction 0 to 4; 5 to 7; 8 to 9; 10 or more years), baseline Clinical Dementia Rating (CDR) score (0 = 0, 0.5 = 1), and NOLAN allocation group (placebo = 0, intervention = 1).

### 2.5 Statistical analyses

We used means and percentages to describe our population. The longitudinal association of MBI domains with biomarkers was assessed using linear mixed‐effects regression models with a random intercept at the individual level. MBI status was coded as absence = 0 and presence = 1 and introduced as a time‐varying variable. The coefficients of the mixed models for domain presence thus express a comparison between those with the presence of the MBI domain (exposure coded = 1) and those without it (reference group coded = 0). Each model used a biomarker as the dependent variable, and the fixed effects were estimated for MBI domains as the main predictor (one domain per model) and for the potential confounding factors: age, sex, time (months), APOE ε4 status, education level, baseline CDR score, and NOLAN allocation group.

A time × MBI interaction was also included as a fixed effect, which is the difference in the rate of change of the biomarker per unit of time, comparing those with present versus absent MBI domain. In other words, if the *p* value of this coefficient is significant, the biomarker displayed faster rates of change in the MBI‐positive than in the MBI‐negative group for that domain. Additionally, to assess the interaction of MBI domains and biomarkers across cognitive function levels, we ran the mixed‐effects models adding an interaction term MBI × CDR × time. Statistical analyses were performed with α = 0.05 in Stata (StataCorp., Stata Statistical Software: Release 17, 2021).

## 3 RESULTS

Our study included 359 participants with a mean age of 78.3 (SD 0.3); the majority were female (60.3%) with high levels of education. Almost two thirds of the study sample showed objective cognitive impairment (MCI) with a CDR score of 0.5. The overall APOE ε4 positivity was 24.3%. One in every three participants was positive for at least one MBI domain at baseline (64.7%), the most frequent being affective dysregulation (50.4%) and impulse dyscontrol (28.5), as presented in Table 1.

**TABLE 1:** Description of study population.

	70 to 79		80+		Total	
*n* (%)	*n* = 219	61.0%	*n* = 140	39.0%	*n* = 359	100%
Age, mean (SD)	75.3	(0.2)	83.0	(0.3)	78.3	(0.3)
Female	132	(60.3)	77	(55.0)	209	(58.2)
Education (years)						
<4 years	3	(1.4)	1	(0.7)	4	(1.1)
4 to 7 years	14	(6.4)	14	(9.9)	28	(7.8)
8 to 9 years	31	(14.2)	29	(20.6)	60	(16.8)
≥10 years	170	(78.0)	97	(68.8)	266	(74.3)
CDR score						
0	98	(44.7)	45	(32.1)	143	(39.8)
0.5	121	(55.3)	95	(67.9)	216	(60.2)
APOE ε4 positive	58	(27.4)	25	(19.8)	83	(24.6)
NOLAN allocation group						
Active	106	(48.4)	72	(51.4)	178	(49.6)
Placebo	113	(51.6)	68	(48.6)	181	(50.4)
MBI domains						
Decreased motivation	24	(11.2)	21	(15.3)	45	(12.8)
Affective dysregulation	107	(50.0)	70	(550.7)	177	(50.3)
Impulse dyscontrol	70	(32.6)	30	(21.9)	100	(28.4)
Social inappropriateness	7	(3.3)	4	(2.9)	11	(3.1)
Abnormal perception	4	(1.9)	2	(1.5)	6	(1.7)
At least one MBI domain positive	119	(66.1)	70	(62.5)	189	(64.7)

The longitudinal associations of the MBI domains and the biomarkers are presented in Table 2. Of the 362 NOLAN participants, 289 had their plasma pTau181 and GFAP measured. Data incompleteness for the covariates resulted in different sample sizes for each model (Table 2).

**TABLE 2:** Longitudinal association of rate of change in plasma biomarkers with MBI domain status and severity.

	MBI domain status (ref = absent)				MBI domain severity (ref = 1‐minimum severity)			
	Coefficient	*p* value	95% CI lb	95% CI ub	Coefficient	*p* value	95% CI lb	95% CI ub
pTAU181, *n* = 271								
Decreased motivation	−0.115	.222	−0.299	0.069	−0.021	0.676	−0.117	0.076
Affective dysregulation	−0.075	.206	−0.191	0.041	−0.026	0.231	−0.069	0.017
Impulse dyscontrol	0.058	.395	−0.076	0.193	0.027	0.336	−0.028	0.082
Social inappropriateness	0.147	.446	−0.231	0.524	0.163	0.093	−0.027	0.353
Abnormal perception	**0.638**	**.023**	**0.087**	**1.188**	**0.427**	**0.006**	**0.125**	**0.729**
GFAp, *n *= 271								
Decreased motivation	0.661	.503	−1.272	2.594	0.449	0.388	−0.571	1.469
Affective dysregulation	−0.608	.352	−1.887	0.672	0.024	0.915	−0.415	0.463
Impulse dyscontrol	0.260	.720	−1.162	1.682	0.056	0.843	−0.493	0.604
Social inappropriateness	−3.050	.135	−7.048	0.948	−1.843	0.072	−3.850	0.165
Abnormal perception	−3.098	.320	−9.207	3.010	−2.873	0.091	−6.204	0.458
Omega‐3, *n* = 329								
Decreased motivation	0.048	.172	−0.021	0.116	0.022	0.233	−0.014	0.059
Affective dysregulation	0.010	.646	−0.033	0.054	−0.004	0.596	−0.020	0.012
Impulse dyscontrol	0.006	.813	−0.043	0.055	0.003	0.762	−0.018	0.024
Social inappropriateness	0.076	.260	−0.056	0.208	0.046	0.179	−0.021	0.113
Abnormal perception	0.000	.998	−0.200	0.200	0.051	0.394	−0.067	0.169
Homocysteine, *n* = 332								
Decreased motivation	−0.104	.196	−0.263	0.054	−0.057	0.180	−0.141	0.026
Affective dysregulation	−0.038	.457	−0.139	0.062	−0.004	0.823	−0.042	0.033
Impulse dyscontrol	−0.005	.937	−0.120	0.111	0.011	0.665	−0.038	0.060
Social inappropriateness	0.010	.951	−0.295	0.314	0.100	0.210	−0.057	0.257
Abnormal perception	**0.570**	**.016**	**0.107**	**1.033**	**0.423**	**0.002**	**0.150**	**0.697**
Ferritin, *n* = 332								
Decreased motivation	−1.841	.100	−4.036	0.354	−1.073	0.070	−2.232	0.086
Affective dysregulation	−0.108	.877	−1.485	1.268	−0.074	0.782	−0.598	0.450
Impulse dyscontrol	−1.124	.163	−2.703	0.454	−0.416	0.232	−1.097	0.266
Social inappropriateness	1.234	.587	−3.215	5.684	0.066	0.954	−2.182	2.315
Abnormal perception	2.378	.492	−4.409	9.165	1.823	0.338	−1.905	5.552
Insulin, *n* = 332								
Decreased motivation	**−1.559**	**.048**	**−3.102**	**−0.015**	−0.747	0.072	−1.561	0.067
Affective dysregulation	−0.480	.335	−1.455	0.495	−0.219	0.242	−0.585	0.148
Impulse dyscontrol	**−1.222**	**.031**	**−2.334**	**−0.110**	−0.441	0.072	−0.922	0.039
Social inappropriateness	−1.629	.291	−4.655	1.396	−0.358	0.651	−1.908	1.193
Abnormal perception	−0.047	.984	−4.717	4.623	0.398	0.771	−2.282	3.078
Transferrin, *n* = 332								
Decreased motivation	0.004	.595	−0.010	0.017	0.000	0.960	−0.007	0.007
Affective dysregulation	0.004	.383	−0.005	0.012	0.002	0.152	−0.001	0.005
Impulse dyscontrol	−0.004	.407	−0.014	0.006	−0.001	0.504	−0.006	0.003
Social inappropriateness	−0.008	.548	−0.034	0.018	−0.003	0.701	−0.016	0.011
Abnormal perception	−0.008	.678	−0.048	0.032	−0.003	0.782	−0.026	0.020
Vitamin D, *n *= 332								
Decreased motivation	0.746	.140	−0.244	1.736	0.382	0.150	−0.138	0.901
Affective dysregulation	0.109	.733	−0.518	0.736	0.079	0.507	−0.154	0.312
Impulse dyscontrol	0.076	.835	−0.643	0.796	0.079	0.612	−0.227	0.386
Social inappropriateness	−0.164	.867	−2.091	1.762	0.043	0.932	−0.948	1.034
Abnormal perception	−0.124	.934	−3.076	2.828	0.316	0.720	−1.411	2.042

*Note*: 95%CI lb = lower bound, ub = upper bound. All models adjusted for age, sex, time, APOE ε4 status, education level, baseline CDR score, NOLAN allocation group.

**FIGURE 1:**
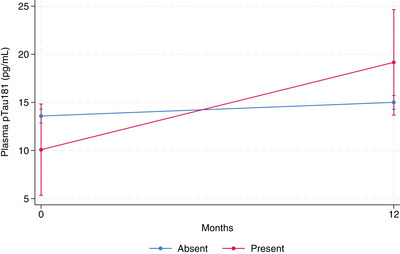
Plasma pTau181 longitudinal change by abnormal perception status. Marginal mean values of biomarker adjusted for age, sex, time, APOE ε4 status, education level, baseline CDR score, NOLAN allocation. Confidence intervals correspond to marginal prediction.

**FIGURE 2:**
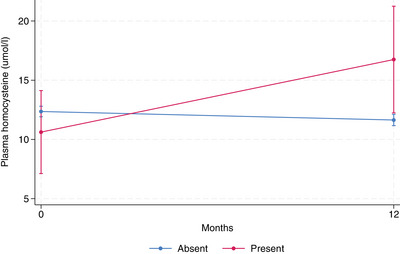
Plasma homocysteine longitudinal change by abnormal perception status. Marginal mean values of biomarker adjusted for age, sex, time, APOE ε4 status, education level, baseline CDR score, NOLAN allocation. Confidence intervals correspond to marginal prediction.

**FIGURE 3:**
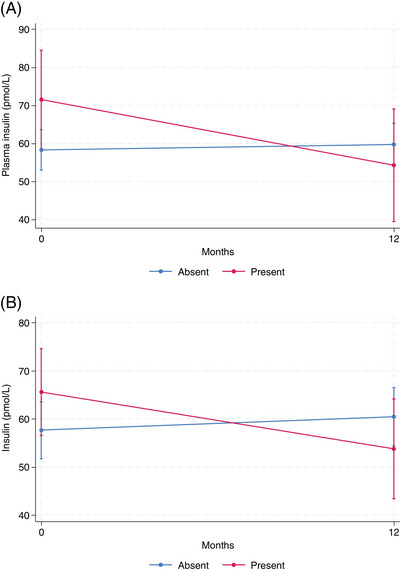
Plasma insulin longitudinal change by MBI status. (A) Decreased motivation status. (B) Impulse dyscontrol status. Marginal mean values of biomarker adjusted for age, sex, time, APOE ε4 status, education level, baseline CDR score, NOLAN allocation. Confidence intervals correspond to marginal prediction.

Significant 1‐year longitudinal associations were found for the following MBI domains and biomarkers:

- pTau181, abnormal perception presence (*p* = 0.023) and its higher severity (*p* = 0.006) were associated with a significantly faster increase in plasma pTau181 (Figure 1).
- Homocysteine, abnormal perception presence (*p* = .015), and higher severity (*p* = .002) were associated with a significantly faster increase in plasma homocysteine (Figure 2).
- Insulin, decreased motivation (*p* = .042), and impulse dyscontrol (*p* = .029). MBI presence was associated with a significantly faster decrease in plasma insulin (Figure 3).

The remaining biomarkers were not associated with MBI domain status or severity over the 1‐year follow‐up.

### 3.1 Exploratory aim

The models including MBI × CDR × time interaction resulted in statistically significant coefficients for the MBI domain of impulse dyscontrol and pTau and between affective dysregulation and the ferritin levels (Table S1).

## 4 DISCUSSION

Our results showed that, after a 1‐year follow‐up, SCD or MCI older adults with the MBI domain of abnormal perception (ie, psychosis, presence, and severity) had steeper increases in plasma pTau181 and homocysteine levels than their counterparts. Also, participants with the presence of decreased motivation and impulse dyscontrol had steeper decreases in insulin than their counterparts.

The MBI domain of psychosis had a high predictive ability for incident dementia in a normal cognition/MCI population^36^ and was associated with APOE ε4 status^37^ and acute medical issues like infection, metabolic disorders, and pain.^17^ We verified if the MBI × APOE ε4 interaction was significant in our data. We found no significant difference in the biomarkers across APOE ε4 status (*p* = .118 for pTau and *p* = .612 for homocysteine); however, this should be interpreted with caution since few participants present with abnormal perception.

Regarding plasma pTau181, researchers have recently found that MBI in both normal cognition/SCD and MCI was cross‐sectionally and longitudinally associated with higher plasma pTau181 levels at baseline, increasing pTau181 over 5 years, a decline in memory and executive function over the same time period, and a higher rate of incident dementia in participants in the ADNI population.^12^ Our results agree with those findings, while adding domain‐specific results as a time‐varying variable in the mixed‐effects models. We consider this an appropriate way of modeling MBI, given the fluctuation of the NPS across the 1‐year interval between NOLAN observations.

On the other hand, the significant interaction for CDR score and impulse dyscontrol (for the MBI‐pTau association) and for affective dysregulation (for the MBI–ferritin association) suggest that these MBI domains could be more prone to be moderated by cognitive function. These exploratory findings are limited by the small sample with MBI positive for the abnormal perception domain, which precluded statistical analysis for this domain.

An analysis of the Swedish BioFINDER2 data evidenced an association of higher MBI‐C scores and higher tau PET deposition in the entorhinal cortex/hippocampus and pTau181 in CSF in Aβ + preclinical AD participants,^38^ as opposed to findings in ADNI,^11^ possibly due to tau PET measurement methods. However, significant associations between MBI and CSF pTau181 at baseline, increasing pTau181 over 4 years, and a higher dementia incidence were reported in ADNI and MEMENTO.^9^ Interestingly, recent findings from the ADNI cohort suggest that amyloid status is a moderator of the association between MBI and pTau uptake in two brain regions of interest. In the latter paper, the authors suggest that MBI is a sequela of neurodegeneration.^15^ Further research is needed to understand better the role and timing of MBI in the natural history of tau pathology in AD.

Our study found that homocysteine levels increased in those with MBI (abnormal perception), regardless of allocation group. This was in contrast to what happened to the average homocysteine levels in NOLAN.^29^ Intriguingly, our results suggest that participants with abnormal perception were refractory to the homocysteine‐lowering effects of the intervention (possibly explained by insufficient levels of vitamin B12 or folate in the intervention or by inadequate enzymatic activity of the methylene tetrahydrofolate reductase due to genetic defects^39^). High plasma homocysteine can present in 10% to 20% of older adults,^40^ with higher prevalence in psychogeriatric patients.^25^, ^41^ The frequency of hyperhomocysteinemia (> 10 μmol/L) was linked to medial temporal lobe atrophy^42^ and with NPS severity (*p* < .0001) and functional impairment.^43^ Additionally, brain structural changes such as medial temporal atrophy (MTA) by computed tomography (CT) scans have been correlated to plasma homocysteine, with more severe MTA associated with higher plasma homocysteine,^42^ with similar findings for diffusion‐tensor imaging (DTI).^44^

The findings regarding hyperhomocysteinemia and abnormal perception might be explained by (1) an indirect mechanism through tau phosphorylation because hyperhomocysteinemia, when caused by low vitamin B12 levels, promotes tau phosphorylation through the regulation of GSK3beta and PP2A^45^ and (2) genotype‐derived enzymatic deficiency given by the A1298C genetic variant of the methylenetetrahydrofolate (MTHFR) reductase.^46^, ^47^

Concerning insulin, participants with the MBI domains of decreased motivation and impulse dyscontrol registered a significant decrease in plasma insulin levels during the 1‐year follow‐up compared to those without these MBI domains. The differences were not explained by fasting/non‐fasting during blood sampling (*p* = .636). The role of brain insulin resistance in neurodegeneration has been proposed to occur via vascular endothelial dysfunction, inflammation, blood–brain barrier injury, white matter disease of vascular origin, and mitochondrial dysfunction, among others,^48^ which is congruent with studies identifying type 2 diabetes as a dementia risk factor.^20^, ^21^ Insulin has shown anti‐depressant‐like activity in murine models, acting, at least partially, via the insulin growth factor 1 (IFG‐1).^49^

A higher frequency of agitation and irritability was reported among diabetic versus non‐diabetic patients with probable AD (*n* = 3569).^50^ Also, impaired insulin signaling as part of allostatic load has been described as a key component of AD.^51^ Therefore, we hypothesize that the impact of impaired insulin signaling in predementia patients could happen through allostatic load as an expression of age‐related insulin secretory dysfunction^52^ mainly in a global way and not specifically for certain MBI domains. This notion is supported by a study that found global MBI status to better predict Aβ42/40 ratio over individual domains.^10^ NOLAN participants with MBI might concurrently have a non‐compensated β‐cell dysfunction and insufficient insulin secretion, as opposed to their counterparts. Further research is warranted to test whether this link is stronger for certain MBI domains or globally.

Omega‐3‐depleted red blood cell membranes and increased GFAP levels have been reported in patients with depression or depressive symptoms.^18^, ^19^, ^22^, ^23^ Still, our study found no association between the omega‐3 index or GFAP concentration and MBI domains, possibly due to the different operationalization of depression (MBI domains vs depressive symptoms). Other metabolic and inflammatory biomarkers have been associated with NPS. Studies have also correlated ferritin/transferrin disruption with NPS, like night agitation and restless leg syndrome.^27^, ^28^ In a psychiatric population with ferritin/transferrin imbalance, iron treatment seemed to have reduced anxiety, irritability, aggressiveness, sadness, anhedonia, apathy, sleep disorders, dysautonomia, eating disorders, and restless leg syndrome, attributable to iron's mono‐aminergic neurotransmitter synthesis activity.^27^ In the same vein, vitamin D has been attributed to several functions in the nervous system (eg, regulation of neurotrophic factor production, neurotransmitter release, calcium homeostasis),^24^ and increased D3 receptor density in the nucleus accumbens and reduced dopaminergic neurotransmission in the amygdala have been linked with psychosis in individuals with AD.^17^ In our study, we did not find a significant association between such biomarkers and MBI domains, possibly due to the early stage of cognitive decline in the NOLAN population.

### 4.1 Strengths and limitations

Our study has several strengths. It is the first study exploring, among older adults with SCD or MCI, MBI associations with a broad scope of plasma biomarkers. Notably, it is the first study to test whether MBI is longitudinally associated with metabolic biomarkers. Yet the study has limitations. The MBI‐C was not used in Nolan. We utilized a previously reported transformation from NPI‐Q items to MBI domains,^5^, ^53^ which are not equivalent but correlated.^54^ To get closer to the ISTAART MBI criteria, we excluded patients with major psychiatric conditions (*n* = 3), but we could not verify that the NPS were later‐life emergent and persistent for at least 6 months (NPI‐Q spans 1 month before the interview). Therefore, our results might be inaccurate due to classification error.

Our findings should be interpreted with caution given the few people positive to the MBI domains of psychosis (*n* = 6) and social inappropriateness (*n* = 11). Despite this, review of the estimates provides reassurance. The coefficient of 0.638 is substantially larger than any of the other estimates, which range from −0.115 to 0.147. Further, the association for psychosis remained significant, despite having the widest 95% confidence interval (likely due to the small sample of six participants). The large effect size of psychosis is also consistent with the literature, which demonstrates strong associations between psychosis and incident dementia.^36^, ^55^, ^56^ A replication of this study with a larger sample size across these domains is certainly necessary to confirm the reproducibility of our results.

Given the secondary analysis nature of our study, we were not able to choose the biomarkers to be analyzed a priori. After reviewing the literature, we decided to use the available biomarkers as a general approach to metabolic dysregulation and links with NPS.

### 4.2 Clinical and research implications

These results are a first approach into the metabolic aspects of NPS. We envision advancing our research with precise biomarkers of organismal and brain‐specific metabolism^57^, ^58^ designing de novo clinical and translational studies that incorporate the MBI‐C scale.

Our results open the door for new hypotheses about the potential benefit of approaching and correcting insulin imbalance and hyperhomocysteinemia in MBI patients. In the same vein, if further studies strengthen the evidence on the MBI–pTau association, pTau‐binding antibodies could be a pharmacological option to treat agitation and psychosis symptoms in the future. New clinical trials should confirm this by assessing pTau‐binding antibodies for agitation and psychosis‐related MBI domains in older adults.^59^

## CONFLICT OF INTEREST STATEMENT

EGB, MM, and AM have nothing to disclose. ZI has served on advisory boards/consultancies for Eisai, Eli Lilly, Lundbeck, Otsuka, Novo Nordisk, Roche, and the Canadian Agencies for Drugs and Technologies in Health; he also serves on the Government of Canada Ministerial Advisory Board for Dementia. He is also supported by the UK National Institute for Health and Care Research Exeter Biomedical Research Centre. BV is an investigator in clinical trials sponsored by Biogen, Lilly, Roche, Eisai, Pfizer, Pierre Fabre Pharmaceuticals, and the Toulouse University Hospital. He has served as SAB member for Biogen, Alzheon, Green Valley, Norvo Nordisk, Longeveron, Rejuvenate Biomed Clinical Pfizer, Eisai France, Advisory Board Meeting—but received no personal compensation. He has served as consultant and/or SAB member for Roche, Lilly, Eisai, TauX, and Cerecin with personal compensation. JD has received payment/honoraria from Biogen (presentation for Biogen in 2021) and has participated on a data safety monitoring board or advisory board for the French board for Roche in 2020 to 2022. MS has served on advisory boards/consultancies Acadia, Otsuka, Avanir, Medesis Pharma, Servier, Eisai, Roche, Biogen, Lilly, and Ethypharm. Author disclosures are available in the supporting information.

## ETHICAL APPROVAL

The NOLAN study (clinicaltrials.gov NCT03080675) was approved by the Advisory Committee for Protection of Persons South West and Overseas II (CPP SOOM II) and by the French Agency for the Safety of Medicines and Health Products (ANSM). All participants signed informed consent.

## FINANCIAL DISCLOSURE

This work has benefited from funding by the Agence Nationale de la Recherche under the France 2030 program (reference number: ANR‐23‐IAHU‐0011).

## CONSENT STATEMENT

The NOLAN study (clinicaltrials.gov NCT03080675) was approved by the Advisory Committee for Protection of Persons South West and Overseas II (CPP SOOM II) and by the French Agency for the Safety of Medicines and Health Products (ANSM). All participants signed informed consent. The study was performed in accordance with the ethical standards as laid down in the 1964 Declaration of Helsinki and its later amendments or comparable ethical standards.

## CLINICAL TRIAL REGISTRY NUMBER

NCT03080675 registered at www.clinicaltrials.gov.

## Supporting information

Supporting Information

Supporting Information
