# Supplementary material for: Mild behavioral impairment domains are longitudinally associated with pTAU and metabolic biomarkers in dementia‐free older adults
Source: Alzheimers Dement. 2024 Jun 14;20(7):4692–701. doi: 10.1002/alz.13902 (PMC11247706; doi:10.1002/alz.13902)
Supplement: Supplementary file 1 — Supporting Information [file ALZ-20-4692-s002.docx]

**Supplementary Table 1. P values of the MBI × CDR × time interactions**

| **MBI domain**  p value for the interaction | **pTau** | **homocysteine** | **omega3** | **GFAP** | **ferritin** | **insulin** | **transferrin** | **vitamin D** |
| --- | --- | --- | --- | --- | --- | --- | --- | --- |
| Decreased motivation | 0.975 | 0.628 | 0.984 | 0.999 | 0.744 | 0.357 | 0.504 | 0.970 |
| Affective dysregulation | 0.284 | 0.847 | 0.323 | 0.757 | **0.017** | 0.335 | 0.558 | 0.274 |
| Impulse dyscontrol | **0.000** | 0.146 | 0.051 | 0.652 | 0.909 | 0.928 | 0.287 | 0.552 |
| Social inappropriateness | 0.756 | 0.425 | 0.637 | 0.598 | 0.955 | 0.618 | 0.796 | 0.769 |
| Abnormal perception | - | - | - | - | - | - | - | - |
| - ommited because all the participants with CDR=1 at baseline were positive for abnormal perception at follow-up | | | | | | | | |

**Supplementary material 1**

Detailed contents of the nutritional blend.

Participants allocated to the active group were instructed to take the nutritional blend daily, for one year. The blend consisted of two soft gel capsules (with 775mg filling each) and by one powdered sachet of ≈15g, to be mixed in 120mL of cold water. Total composition of a daily dose of the NB consisted of 50mg of thiamin, 15 mg of riboflavin, 25mg of niacin, 23mg of pantothenic acid, 18mg of pyridoxine, 0.15mg of biotin, 0.4mg of folic acid, 0.5mg of cobalamin, 82.6mg of vitamin E, 500mg of vitamin C, 15μg of Vitamin D, 85mg of choline, 80μg of selenium, 3g of citrulline, 700mg of EPA and 770mg of DHA.

Active soft gel capsules provided 125 mg of EPA and 250 mg of DHA from vegetal origin. The rest of nutrients were given under their tolerable upper intake level (UL) in the powdered drink mix.

Placebo soft gel capsules contained a mixture of vegetable oils free of EPA and DHA, but with a similar profile in fatty acids as the active capsules. The placebo powdered drink did not contain any of the active ingredients but was matched for carbohydrate and protein content to the active powdered drink. It was composed of sucrose / starch, polydextrose, proteins, flavors, natural colorant and sweeteners, to replicate taste, texture and appearance of the active powdered drink.
